# Patient-proxy agreement on health-related quality of life in juvenile fibromyalgia syndrome

**DOI:** 10.1186/s12969-019-0320-y

**Published:** 2019-05-09

**Authors:** Sabrina Gmuca, Rui Xiao, David D. Sherry

**Affiliations:** 1 Department of Pediatrics, Division of Rheumatology, Children’s Hospital of Philadelphia, 34th St and Civic Center Blvd, Philadelphia, PA 19104 USA; 2 Center for Pediatric Clinical Effectiveness, Children’s Hospital of Philadelphia, 2716 South Street, Philadelphia, PA 19146 USA; 3 PolicyLab, Children’s Hospital of Philadelphia, 2716 South Street, Philadelphia, PA 19146 USA; 4 Department of Biostatistics, Epidemiology & Informatics, Perelman School of Medicine at The University of Pennsylvania, Philadelphia, USA; 5 Children’s Hospital of Philadelphia, Roberts Center for Pediatric Research, 2716 South Street, 11214, Philadelphia, PA 19146 USA

**Keywords:** Health-related quality of life, Juvenile fibromyalgia syndrome, Adolescent, Chronic musculoskeletal pain syndromes

## Abstract

**Background:**

Health-related quality of life (HRQoL) measures serve as important indicators of pain-related physical and psychosocial function in youth with juvenile fibromyalgia syndrome (JFMS). While the administration of parent-proxy reported HRQoL measures in the assessment of JFMS is common, its added clinical value to patient self-reports is unclear. We aimed to determine the level of agreement on HRQoL among patients with JFMS as well as their parent-proxies and to determine factors associated with this agreement.

**Methods:**

We performed a retrospective, cross-sectional cohort study of children aged 8 to 17 years diagnosed with JFMS and presenting for initial evaluation to a pediatric rheumatology pain clinic between April 2017 and May 2018. All patients and proxies were administered the Pediatric Quality of Life Short Form 15 Generic Core Scales (PedsQL SF-15) as part of routine clinical care. We calculated absolute discrepancy scores (absolute value of parent-proxy score minus patient score) to describe the extent of difference in HRQoL scores between parent-proxies and patients. We examined agreement between parent-proxy report and patient self-report via intraclass correlation coefficients (ICCs), stratified by age and sex, as well as Bland-Altman plots. We also used multivariate regression models to determine factors associated with level of agreement.

**Results:**

A total of 65 patient-proxy pairs were included in this study. ICCs demonstrated good to excellent agreement between all parent-proxy and patient measures of HRQoL irrespective of the patient’s age or sex. The level of agreement was not associated with pain duration or pain severity but less agreement on psychosocial HRQoL was associated with older patient age (β = 1.30; *p* < 0.05).

**Conclusions:**

This study in youth with JFMS demonstrated good to excellent patient-proxy agreement across all domains of the PedsQL SF-15 irrespective of patient’s age or sex. Our findings suggest that parent-proxy reports do not provide additional information beyond that obtained from the patient self-report of HRQoL according to the PedsQL SF-15. In order to facilitate children and adolescents with JFMS becoming partners in their own healthcare, and to decrease the burden of multiple questionnaires, we propose focusing on patients’ own perceptions of HRQoL in the clinical setting.

## Background

Health-related quality of life (HRQoL) is a multidimensional concept reflecting the impact of disease and treatment on a patient’s subjective assessment of his or her functioning and emotional wellbeing [1]. Patient and parent-proxy reported HRQoL measures serve as important measures of pain-related physical and psychosocial function in children and adolescents with juvenile fibromyalgia syndrome (JFMS) [2]. However, overutilization of patient-reported outcome measures (PROs) can result in questionnaire fatigue for patients and their caregivers. Therefore, judicious use of these measures is warranted in order to optimize clinical care for patients with JFMS and their families [3].

JFMS is one of the most common pediatric chronic musculoskeletal pain conditions, affecting approximately 1 in 20 children [4] and predominantly adolescent girls [5–9]. Up to 25% of new referrals to pediatric rheumatology are for non-specific musculoskeletal pain [10]. JFMS is a chronic disorder characterized by widespread musculoskeletal pain in combination with a number of somatic symptoms including fatigue, nonrestorative sleep, and sensory, autonomic, and cognitive dysfunction [11–13]. JFMS is associated with significant morbidity and impaired quality of life for affected children and their families [14–16]. Children with JFMS have high rates of school absenteeism [17] and psychological co-morbidities, including anxiety, depression, and post-traumatic stress disorder [18–21]. Family members of adolescents with JFMS are also affected by the child’s pain [22, 23]. Specifically, mothers of children with chronic pain have been found to have poor health-related quality of life (HRQoL), which correlates significantly with their child’s pain severity [24]. This is likely related to the increased burden of caring for a child both emotionally and financially, as parents must take off from work to bring their child to appointments and care for them during school absences. Therefore, limiting the burden of patient and parent-proxy questionnaires is important in reducing the over medicalization of children with JFMS and has implications for caregivers’ quality of life as well.

For patient-reported outcome measures (PROs), patient self-report and parent-proxy report provide important perspectives on pain and health outcomes. The Pediatric Quality of Life Inventory, Version 4.0 Generic Core Scales (PedsQL) Short Form 15 (PedsQL SF-15), has been used widely in both healthy and non-healthy populations to assess HRQoL in children and adolescents. It has demonstrated good construct validity, predictive validity, and responsiveness. A number of studies evaluating agreement between patient and parent-proxy ratings of HRQoL using the PedsQL have found positive correlations in samples of children with medical problems [25–31]. There has been one study examining patient and parent-proxy agreement among youth with JFMS, which showed moderate to good agreement [2]. However, this study did not examine the factors that influence the level of agreement (including pain duration and pain severity) and whether this level of agreement is true irrespective of patient age or sex.

While the administration of parent-proxy reported HRQoL measures in the assessment of JFMS is common, its added clinical value to patient-reported measures is unclear. Our study aimed to characterize agreement between parent-proxy reports and patient self-reports of HRQoL in a population of children and adolescents with JFMS by 1) evaluating discrepancy between parent-proxy reports and patient self-reports of HRQoL, 2) determining concordance between parent-proxy reports and patient self-reports of HRQoL and 3) examining this concordance stratified by patient age and sex. We also aimed to examine potential factors influencing patient-proxy agreement. We hypothesized that there would be good agreement between parent-proxy reports and patient self-reports of HRQoL and that patient age, duration of pain, and pain severity would be associated with this agreement.

## Methods

### Study population

We performed a retrospective, cross-sectional cohort study of children aged 8 to 17 years who presented for an initial evaluation to a pediatric rheumatology pain clinic at a tertiary care hospital from April 2017 to May 2018, and one of their parents or legal guardians. All patients and proxies were administered the PedsQL SF-15 as part of routine clinical care. This was only available in English. Online questionnaires were administered separately to patients and their proxies at the time of the clinic visit. For the purposes of this study, we utilized data (including PedsQL SF-15 responses) from the time of the initial clinic visit only (no follow-up data were utilized). Families were approached for consent (and/or assent) to enroll in an existing institutional review board (IRB)-approved registry of patients with chronic musculoskeletal pain, with approximately < 1% of new patients declining inclusion in this registry. We included patients meeting the following criteria: 1) enrolled in the patient registry, 2) completed the PedsQL SF-15, 3) had a parent-proxy complete the PedsQL SF-15 and, 4) fulfilled the 2010 American College of Rheumatology (ACR) criteria for fibromyalgia syndrome [32]. Additional information collected in the patient registry included patient age, sex, race, ethnicity, pain severity, and duration of pain (months). This study received exemption from the study site’s IRB.

### Clinical characteristics

Clinical and demographic information for this study were abstracted and analyzed from an existing prospective patient registry, or research database, for the pediatric rheumatology pain clinic. This prospective patient registry includes data from the initial clinic visit and all subsequent follow-up visits for patients seen in the specialty pain clinic. It is periodically updated and maintained to include variables of interest by multiple research assistants staffed within the clinic. Demographics and clinical characteristics documented for each initial visit include age, race, ethnicity, sex, past medical, psychological and surgical history, past medications, social information, and educational information. All clinic visits (initial and follow-up visits) include information regarding physical exam findings, current medications, pain severity and quality, and diagnosis and treatment recommendations. Additionally, all visits include measures that are part of routine clinical care including the patient- and parent-proxy reported Functional Disability Inventory (FDI) [31, 33] scores, pain visual analog scale (VAS) (0–100), verbal pain report (0–10), symptom severity score (SSS; 0–12), and widespread pain index (WPI; 0–19). Lastly, the presence and number of adverse childhood experiences (ACEs) (reported at the time of the initial clinic visit only) are documented. ACEs are potentially traumatic childhood events that can have negative, lasting effects on health and well-being [34]. Patients were considered to have a positive history for ACEs if they had been exposed to any of the following: 1) verbal abuse, 2) physical abuse, 3) sexual abuse, 4) parent with an alcohol problem, 5) parent with a drug problem, 6) parental divorce, 7) parental separation, 8) other household members with problems with drugs and/or alcohol, 9) household mental illness/suicide, 10) incarcerated household member, 11) economic hardship, 12) mother or step mother is a victim of domestic violence, and 13) bullying [35]. All data used for the purposes of this study were solely from the first documented clinic encounter.

### HRQoL measures

Patients and one of their parents reported the child’s HRQoL using the PedsQL SF-15, which is a tool consisting of 15 items that assesses patients on 4 domains: physical functioning, emotional functioning, social functioning, and school functioning [34]. Children report problems performing in these domains over the past 7 days using a Likert-type scale, with 0 indicating “never a problem” to 4 indicating “almost always a problem.” Response to each item is reverse scored and linearly transformed to a 0–100 point scale (0 = 100, 1 = 75, 2 = 50, 3 = 25, 4 = 0). Responses in each domain are averaged for Total HRQoL, and 2 summary scores (Physical Health and Psychosocial Health), with higher scores indicating better HRQoL. The Physical Health Summary score consists of scores from the physical functioning domain, and the Psychosocial Health summary score consists of scores from the emotional, social and school functioning domains. The PedsQL SF-15 has been validated in children ages 8–17 years and their parents [26, 36].

### Statistical analyses

We calculated Cronbach’s alpha coefficients to estimate the internal consistency of the items in the HRQoL and assumed a minimum standard of 0.70 as adequate. We calculated absolute discrepancy scores (absolute value of parent-proxy score minus patient score) to describe the extent of difference in HRQoL scores between parent-proxies and patients. A greater discrepancy between parent-proxy report and self-report was indicated by higher scores. We created histograms of the raw differences between patient and parent-proxy scores to demonstrate the magnitude and direction of discrepancies between parent-proxies and patients.

We first examined the agreement between parent-proxy report and patient self-report of HRQoL using intraclass correlation (ICC) and the 95% confidence interval (CI), which were calculated based on a mean-rating, absolute-agreement, 2-way mixed-effects model. ICC cutoffs were interpreted as previously defined: ≤0.4, poor to fair agreement; 0.41 to 0.60, moderate agreement; 0.60 to 0.80, good agreement; and 0.81 to 1.00, excellent agreement [37, 38]. We also assessed the agreement graphically by Bland-Altman plots. We performed multivariate regression models to explore the effect of different independent variables (sex, age, pain duration, pain severity) on the relationship between patient and parent-proxy reports with the absolute value of discrepancy scores for HRQoL serving as the outcome variable in these models. We also used the likelihood ratio (LR) test to determine whether univariate linear regression analyses of patient and parent-proxy scores for each domain of HRQoL were statistically significantly different from adjusted models (including sex, age, pain duration (months), and verbal reported pain score (0–10)). *P*-values less than 0.05 were considered statistically significant. All statistical analyses were performed using statistical software, StataCorp 15 (College Station, TX).

## Results

During the study interval, 215 patients were evaluated in the pediatric rheumatology pain clinic and consented to enrollment in the patient registry. Of these, 7 were missing both the WPI and SSS and 2 subjects were missing the SSS. Of the remaining 206 subjects, 35.4% (*n* = 71) met the 2010 ACR criteria for fibromyalgia syndrome. Notably, 3 subjects had pain for less than 3 months and 2 were diagnosed with complex regional pain syndrome. There were 6 subjects with missing information for the PedsQL SF-15 (one subject was missing both patient and parent-proxy measures; 2 were missing patient reports only; and 3 were missing parent-proxy reports only). Thus, we had 65 evaluable subjects.

Table 1 shows patient demographics and clinical characteristics. Patients were predominantly female (87.7%; *n* = 57) and non-Hispanic Caucasians (80.6%; *n* = 50). The median age of patients at presentation was 15 years (IQR: 14, 16) and the median reported duration of pain prior to clinic evaluation was 24 months (IQR: 12, 48). Patients had moderate pain at presentation according to both the verbal pain score (median 5 [IQR: 4, 7)]) and pain visual analog scale (median 59 [IQR: 42, 74]) [39]. The functional disability score was a median of 25 (IQR: 18, 33), corresponding to moderate disability. According to self-report, psychological co-morbidities were common with 64.6% reporting a history of anxiety and/or panic attacks. Depression was reported in 38.5% of subjects and 33.9% reported suicidality. Adverse childhood experiences were prevalent, with at least one ACE reported in 66.2% of patients at the time of their initial clinic evaluation. The most common co-morbid medical conditions included asthma (36.9%), atopic dermatitis (15.4%), and attention deficit disorder (ADD)/ attention deficit hyperactivity disorder (ADHD) (10.8%). One patient was noted to have benign hypermobility syndrome and 2 patients had gastroesophageal reflux disease. Rheumatologic co-morbidities included inflammatory bowel disease (*n* = 2), juvenile idiopathic arthritis (*n* = 2), autoimmune thyroid disease (*n* = 2), psoriasis (*n* = 2), vasculitis (*n* = 2), and enthesitis (*n* = 1). Data recording parents’ marital status were available. Parents were mostly married (62.5%), with divorce reported in 20.3% (*n* = 13) and marital separation in 6.3% (*n* = 4).

**Table 1:** Patient Demographics and Clinical Characteristics (*n* = 65)

Demographic and Clinical Characteristics	Total *n* = 65
Female, n (%)	57 (87.7)
Age (years), median (IQR)	15 (14, 16)
Non-Hispanic white, n (%)^a^	50 (80.6)
Pain duration (months), median (IQR)	24 (12, 48)
Verbal pain (0–10), median (IQR)	5 (4, 7)
Pain visual analog scale, median (IQR)	59 (42,74)
Functional disability inventory (FDI) (0–60), median (IQR)	25 (18, 33)
Widespread pain index (WPI) (0–19), median (IQR)	11 (9, 14)
Symptom Severity Score (SS score) (0–12), median (IQR)	7 (5, 10)
Reported energy level (0–100%), median (IQR)	60 (40, 70)

*IQR* interquartile range.

^a^Ethnicity and/or race was missing for 3 subjects

**Fig. 1:**
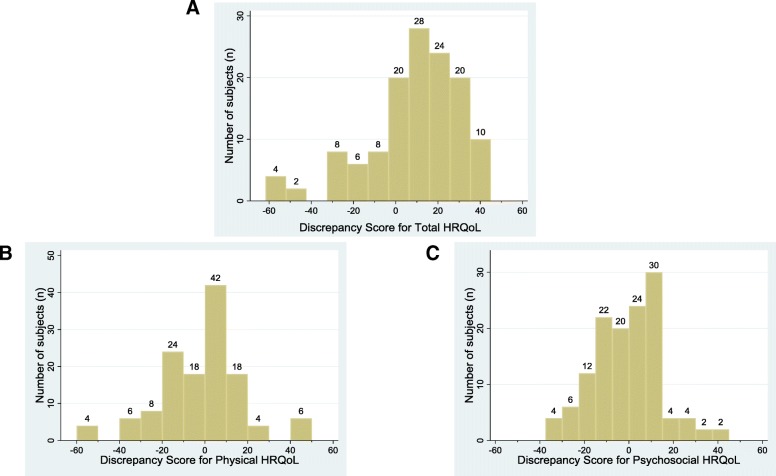
Distribution of raw discrepancy scores between parent proxy report and child self-report of (**a**) Total, (**b**) Physical and (**c**) Psychosocial health-related quality of life (HRQoL)

Table 2 summarizes the absolute discrepancies for HRQoL scores for the entire cohort and stratified by age and sex. Mean parent-proxy and patient scores of total HRQoL were 49.6 (SD 15.7) and 52.3 (SD 18.3), respectively with a mean absolute difference of 20 points (SD 14.0). The mean discrepancy scores for the Physical HRQoL and Psychosocial HRQOL scores were 15.0 (SD 14.3) and 12.1 (SD 9.9) respectively. Figure 1 provides a visual representation of the raw discrepancies between parent-proxy and patient report for Total, Physical and Psychosocial HRQoL.

**Table 2:** Parent-Proxy and Patient Reported HRQoL Scores (According to the PedsQL SF-15)

Characteristic	N	Parent-Proxy Score, Mean (SD)	Patient Score, Mean (SD)	Discrepancy Score, Mean (SD)
Total HRQoL				
All subjects	65	49.5 (15.7)	52.3 (18.3)	20.0 (14.0)
Female	57	49.9 (14.9)	52.1 (17.8)	20.6 (13.9)
Male	8	46.5 (21.5)	54.0 (22.9)	15.6 (15.2)
8–12 years	11	49.2 (17.2)	53.2 (20.1)	16.4 (14.9)
13–18 years	54	49.6 (15.6)	52.2 (18.2)	20.7 (13.9)
Physical HRQoL				
All subjects	65	25.7 (21.1)	40.2 (24.9)	15.0 (14.3)
Female	57	35.7 (20.0)	39.6 (23.8)	15.1 (14.2)
Male	8	35.6 (30.0)	45.0 (33.1)	14.4 (16.1)
8–12 years	11	49.5 (23.2)	50.5 (26.0)	17.3 (14.0)
13–18 years	54	32.9 (19.8)	38.2 (24.3)	14.5 (14.4)
Psychosocial HRQoL				
All subjects	65	56.6 (18.3)	58.4 (21.0)	12.1 (9.9)
Female	57	57.24 (17.8)	58.4 (21.4)	12.2 (9.6)
Male	8	51.9 (21.7)	58.4 (19.6)	11.6 (12.6)
8–12 years	11	49.1 (19.2)	54.5 (19.9)	9.1 (11.5)
13–18 years	54	58.1 (17.8)	59.2 (21.3)	12.7 (9.5)
Emotional Functioning HRQoL				
All subjects	65	51.3 (21.5)	54.6 (23.9)	15.1 (14.5)
Female	57	50.9 (21.0)	54.3 (23.7)	15.7 (14.8)
Male	8	54.7 (26.5)	56.3 (26.9)	10.9 (11.9)
8–12 years	11	44.9 (22.7)	51.7 (18.3)	14.8 (13.2)
13–18 years	54	52.7 (21.3)	55.1 (24.9)	15.2 (14.8)
Social Functioning HRQoL				
All subjects	65	72.1 (26.1)	77.1 (26.1)	16.0 (14.8)
Female	57	72.8 (26.0)	76.9 (27.0)	15.8 (14.2)
Male	8	66.7 (27.8)	78.1 (18.9)	17.7 (19.1)
8–12 years	11	60.6 (23.9)	65.9 (28.2)	17.4 (16.0)
13–18 years	54	74.4 (26.0)	79.3 (25.3)	15.7 (14.6)
School Functioning HRQoL				
All subjects	65	47.6 (25.2)	44.4 (25.7)	18.1 (14.8)
Female	57	49.6 (25.0)	44.8 (26.1)	18.0 (14.8)
Male	8	33.3 (23.6)	41.7 (24.0)	18.8 (15.9)
8–12 years	11	43.2 (22.9)	47.0 (25.3)	15.9 (13.7)
13–18 years	54	48.5 (25.8)	43.9 (25.9)	18.6 (15.2)

Legend. Discrepancy score is the mean of the absolute value of parent score minus child score. HRQoL indicates health-related quality of life (Pediatric Quality of Life Inventory, Version 4.0 Generic Core Scales [PedsQL]; 0–100; higher scores indicate better HRQoL

**Fig. 2:**
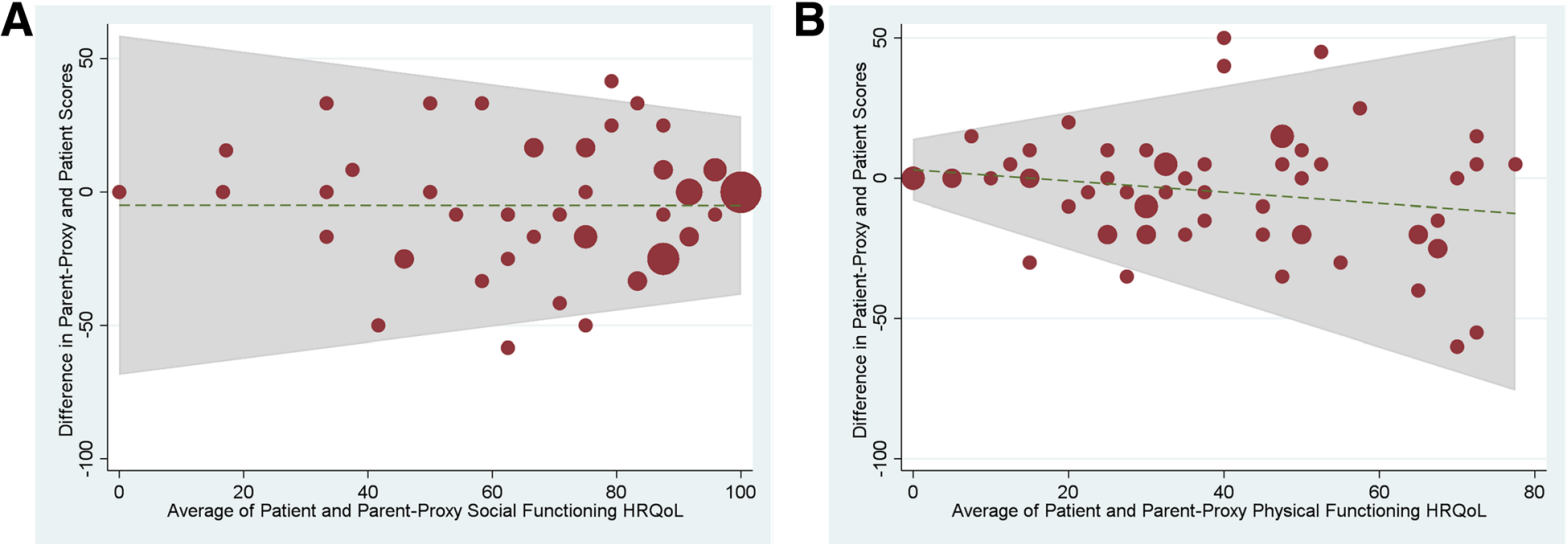
Bland Altman Plots showing means of and difference between child and parent health-related quality of life scores for **a**) Social Functioning HRQoL and **b**) Physical HRQOL. The diameter or width for each plot, represents number of dyads with indicated difference (y-axis) at specified level of HRQoL (x-axis)

Table 3 lists the ICCs for parent-proxy and patient HRQoL scores, demonstrating good to excellent agreement between all parent-proxy and patient measures of HRQoL. Figure 2 includes Bland-Altman plots showing differences between patient and parent-proxy reports at different levels of HRQoL. Interestingly, these plots show a trend towards greater agreement on Social Functioning for higher average levels of Social Functioning, whereas greater patient-proxy agreement on Physical Functioning is demonstrated for lower average levels in this domain of HRQoL.

**Table 3:** Level of Patient-Proxy Agreement (ICCs) on PedsQL SF-15 Scores (*n* = 65)

PedsQL Domain		Patient-Proxy ICC	
		ICC	95% CI
Physical Functioning			
All subjects	65	0.75	0.60–0.85
Female	57	0.72	0.53–0.83
Male	8	0.88	0.47–0.98
8–12 years	11	0.74	−0.02 – 0.93
13–18 years	54	0.74	0.55–0.85
Emotional Functioning			
All subjects	65	0.73	0.57–0.84
Female	57	0.70	0.50–0.82
Male	8	0.90	0.50–0.98
8–12 years	11	0.72	0.04–0.92
13–18 years	54	0.74	0.55–0.85
Social Functioning			
All subjects	65	0.79	0.66–0.87
Female	57	0.81	0.68–0.89
Male	8	0.67	−0.47 – 0.92
8–12 years	11	0.75	0.08–0.93
13–18 years	54	0.79	0.64–0.88
School Functioning			
All subjects	65	0.73	0.57–0.84
Female	57	0.74	0.56–0.85
Male	8	0.69	−0.57 – 0.93
8–12 years	11	0.77	0.14–0.94
13–18 years	54	0.73	0.54–0.84
Psychosocial Health Summary Score			
All subjects	65	0.82	0.70–0.89
Female	57	0.82	0.69–0.89
Male	8	0.81	0.18–0.96
8–12 years	11	0.85	0.48–0.96
13–18 years	54	0.80	0.66–0.89
Total Summary Score			
All subjects	65	0.79	0.66–0.87
Female	57	0.78	0.63–0.87
Male	8	0.85	0.33–0.97
8–12 years	11	0.83	0.38–0.95
13–18 years	54	0.78	0.63–0.87

Legend. PedsQL SF-15 = pediatric quality of life inventory short form 15 item. HRQoL = health-related quality of life. PedsQL SF-15 scores range from 0 to 100 with higher scores indicating better HRQoL. Intra-class correlations (ICCs) were rated as 0–0.40: poor agreement; 0.41–0.60: moderate agreement; 0.61–0.80: good agreement; and 0.81–1.00 excellent agreement. The Psychosocial Health Summary Score is the sum of the items over the number of items answered in the Emotional, Social, and School Functioning Scales. The Physical Health Summary score is equal to the Physical Functioning Scale Score. The Total Summary Score is the sum of all items over the number of items answered on all of the scales

Regression coefficients for the absolute difference in patient-proxy scores were similar in models adjusting for age, sex, pain duration and pain severity for all domains of HRQoL except for psychosocial HRQoL. In this regression model, older age (β = 1.30; *p* < 0.05) was independently associated with less agreement between patient self-report and parent-proxy report. Pain duration and pain severity were not significantly associated with the level of agreement between patients and parent-proxies.

## Discussion

This study, examining the level of agreement on HRQoL among patients with JFMS and their proxies, demonstrated good to excellent agreement across all domains of the PedsQL SF-15 irrespective of patient’s age or sex. Furthermore, pain severity and pain duration did not influence patient-proxy agreement. Older patient age, however, was associated with less agreement between patient self-report and parent-proxy report for psychosocial HRQoL. Our findings suggest that parent-proxy reports serve as valid substitutions for patient self-reports but do not provide additional information beyond that obtained from the patient self-report of HRQoL according to the PedsQL SF-15. In order to facilitate children and adolescents with JFMS becoming partners in their own healthcare, and to decrease the burden of multiple questionnaires, we propose focusing on patient’s own perceptions of HRQoL in the clinical setting.

Our study findings are in line with previous studies assessing agreement between patient and parent-proxy reports of HRQoL using the PedsQL SF-15 [2, 26–31, 40, 41]. In fact, in comparison to the study findings specific to a population of youth with fibromyalgia syndrome [2], we found even greater patient-proxy agreement, according to ICCs, for all domains of HRQoL. Given that the majority of patients with JFMS are females and adolescents, it is important to know whether this agreement holds true for male patients as well as for patients in early childhood. Our study demonstrated continued good to excellent patient-proxy agreement on HRQoL even when stratifying by patient age and biological sex.

This study has limitations. Our sample was restricted to children and adolescents seen in a specialized pediatric rheumatology pain clinic at a tertiary care hospital and therefore may not be reflective of the general pediatric population of patients with JFMS. However, our findings are similar to those previously reported in another sample of youth with JFMS [2] and measures of disease severity, such as pain duration and pain intensity, did not influence patient-proxy agreement. Another limitation was that we did not have detailed data on the proxies, specifically the proxies’ medical histories or socioeconomic status, to include in our analyses. We did not know the proxies’ relationship to the patient (e.g. mother, father, etc.) and therefore, we could not assess for the influence of proxy relationship on patient-proxy agreement. Patients with intellectual disabilities, non-English speaking patients, and patients younger than 8 years old were excluded and our findings cannot be applied to these populations although the PedsQL SF-15 is not validated for children < 8 years. Furthermore, even though patients and proxies were instructed to complete the online questionnaires separately on their own tablets, they may have influenced each other’s responses since they completed the questionnaires in each other’s presence at the time of the clinic visit. However, this minimized the likelihood of proxies completing the patient version of the questionnaire, which would have been a possible source of cross-contamination if families were requested to complete questionnaires prior to the clinic visit. Lastly, this study did not include a qualitative component in that we did not directly ask patients or their proxies as to whether they felt that completion of multiple questionnaires was, in fact, burdensome. Future research might consider patient and parent-proxy surveys on perceptions of questionnaire fatigue.

This study has significant strengths. First, we leveraged the existence of a large patient registry reflecting real world data in a clinical setting. Second, this study is strengthened by the use of multiple statistical methods to characterize and assess patient-proxy agreement on HRQoL according to the PedsQL SF-15. All of our methods demonstrated good patient-proxy agreement, highlighting the robust nature of our findings. By using statistical tests of agreement with absolute discrepancy scores, histograms, ICCs and Bland-Altman plots, our study provides a comprehensive evaluation of patient-proxy agreement on HRQoL [25, 42]. We also employed regression modeling to determine factors influencing patient-proxy agreement and found that pain severity and pain duration did not influence this agreement. However, older patient age was associated with greater patient-proxy disagreement for psychosocial HRQoL and further emphasizes the importance of assessing the patient’s self-report over the parent-proxy’s report. Future studies should consider applying mixed-methods research to gain better insight regarding the factors that contribute to patient-proxy disagreement.

## Conclusions

In summary, we found good patient-proxy agreement on HRQoL in a population of children and adolescents with JFMS at time of initial presentation to a pediatric rheumatology pain clinic. These findings were consistent irrespective of patient age or sex and were not dependent on duration of symptoms or pain severity, highlighting that patients are goodreporters of their HRQoL and youth with JFMS communicate well with their caregivers. We recommend, when possible, administering the PedsQL SF-15 to patients only to minimize the burden of questionnaires in the clinical setting. Future research should explore whether patient-proxy agreement on HRQoL changes with time over the patient’s clinical course and in response to treatment.

## Abbreviations

ACE: Adverse childhood experience
ACR: American College of Rheumatology
CI: Confidence interval
FDI: Functional disability inventory
HRQoL: Health-related quality of life
ICC: Intraclass correlation coefficient
IQR: Interquartile range
IRB: Institutional review board
JFMS: Juvenile fibromyalgia syndrome
PedsQL SF-15: Pediatric Quality of Life Short Form 15 Generic Core Scales
PRO: Patient-reported outcome
SSS: Symptom severity score
VAS: Visual analog scale
WPI: Widespread pain index
